# Gut Bacterial Communities in the Giant Land Snail *Achatina fulica* and Their Modification by Sugarcane-Based Diet

**DOI:** 10.1371/journal.pone.0033440

**Published:** 2012-03-15

**Authors:** Alexander M. Cardoso, Janaína J. V. Cavalcante, Ricardo P. Vieira, Joyce L. Lima, Maria Angela B. Grieco, Maysa M. Clementino, Ana Tereza R. Vasconcelos, Eloi S. Garcia, Wanderley de Souza, Rodolpho M. Albano, Orlando B. Martins

**Affiliations:** 1 Instituto Nacional de Metrologia, Qualidade e Tecnologia, Rio de Janeiro, Brazil; 2 Instituto de Bioquímica Médica, Universidade Federal do Rio de Janeiro, Rio de Janeiro, Brazil; 3 Instituto Nacional de Controle da Qualidade em Saúde, Fundação Oswaldo Cruz, Rio de Janeiro, Brazil; 4 Laboratório Nacional de Computação Científica, Rio de Janeiro, Brazil; 5 Instituto Oswaldo Cruz, Fundação Oswaldo Cruz, Rio de Janeiro, Brazil; 6 Instituto de Biofísica Carlos Chagas Filho, Universidade Federal do Rio de Janeiro, Rio de Janeiro, Brazil; 7 Departamento de Bioquímica, Universidade do Estado do Rio de Janeiro, Rio de Janeiro, Brazil; American University in Cairo, Egypt

## Abstract

The invasive land snail *Achatina fulica* is one of the most damaging agricultural pests worldwide representing a potentially serious threat to natural ecosystems and human health. This species is known to carry parasites and harbors a dense and metabolically active microbial community; however, little is known about its diversity and composition. Here, we assessed for the first time the complexity of bacterial communities occurring in the digestive tracts of field-collected snails (FC) by using culture-independent molecular analysis. Crop and intestinal bacteria in FC were then compared to those from groups of snails that were reared in the laboratory (RL) on a sugarcane-based diet. Most of the sequences recovered were novel and related to those reported for herbivorous gut. Changes in the relative abundance of *Bacteroidetes* and *Firmicutes* were observed when the snails were fed a high-sugar diet, suggesting that the snail gut microbiota can influence the energy balance equation. Furthermore, this study represents a first step in gaining a better understanding of land snail gut microbiota and shows that this is a complex holobiont system containing diverse, abundant and active microbial communities.

## Introduction

The giant African snail *Achatina fulica* is native to the forest areas of East Africa but due to human interference and to its high invasive capacity it can nowadays be found in many regions around the globe including rainforests in Brazil. This mollusk has been reported to be an intermediate vector of worms and microorganisms, causing a wide variety of diseases [1], [2]. *A. fulica* is highly adaptable to a broad range of environments, possibly modifying its gut microbiota to suit local conditions. Therefore, it has been suggested that terrestrial species have a great capacity of adaptation and survival and may contain an intriguing microbiota that specializes in the rapid hydrolysis and fermentation of lignocellulosic plant biomass with an extraordinary efficiency [3]. However, microbial diversity within pulmonate land snails has so far been only poorly investigated by earlier studies using cultivation methods [3], [4].

Recent metagenomic and *in silico* studies have provided strong evidence that gut bacteria perform useful functions to the host, such as digestion of complex polysaccharides, generation of energy (converting sugars into short-chain fatty acids), synthesis of essential amino acids and vitamins, prevention of growth of harmful organisms, and defense against some diseases [5]–[7]. Studies using invertebrates also suggest that there is much microbial diversity yet to be described that could reveal interesting metabolic interactions. For example, the first metagenomic analysis of the hindgut microflora of a higher termite shed light on the microbial metabolism and relevant functional genes for biotechnological applications, such as biofuel production [8]; in bloodfeeding invertebrates such as the medicinal leech, complex microbial communities are probably important for host fitness because of the need for blood-scarce nutrients [9]; in tsetse flies and mosquitoes their associated microbiota may also influence on insect host vector competence by several mechanisms such as activating their immune responses or directly inhibiting pathogen development [10].

Recent work shows that the planorbid snails contain a highly diverse gut bacterial community [11] but to date we know relatively little about the microbiota associated with land snails. Herein, to assess the microbial diversity and succession of the bacterial community in the gastrointestinal tract of the invasive giant land snail, a culture-independent molecular analysis was performed. This study represents the first investigation that reveals the gut bacterial communities within *Achatina fulica*, and also compares the effect of the sugarcane-based diet on gut community composition.

## Materials and Methods

### Ethics statement

An ethics statement is not required for this work. No specific permits were required for the described field studies. The location is not privately-owned or protected in any way and did not involve endangered or protected species.

### Recovery of bacteria from crop and fecal samples

Seven field-collected *Achatina fulica* snails weighing in the range of 70–80 g were captured in Rio de Janeiro, Brazil. The freshly collected fecal samples were placed in a sterile tube on ice and immediately transferred to the laboratory and frozen at −80°C until DNA extraction. To minimize the occurrence of transient bacteria within the crop fluid, the snails were kept under starvation conditions with a natural photoperiod for approximately 72 h, without water and with no substrate before sample collection by cannulation of the mouth-oesophagus with a needleless scalp vein set attached to a syringe (Figure S1). The same animals were then lab-reared. Their diets consisted of a high energy meal diet containing only sugarcane for six months. From these, three grams of pooled fecal samples were suspended vigorously in a 50-ml Falcon tube that contained 45 ml of PBS (phosphate-buffered saline; 8 g.l^−1^ NaCl, 0.2 g.l^−1^ KCl, 1.44 g.l^−1^ Na_2_HPO_4_·12H_2_O, 0.24 g.l^−1^ KH_2_PO_4_, pH 7.6). The fecal suspension and crop fluid were then filtered through 0.2 µm Sterivex filters (Millipore) after filtration through 3.0 µm to separate free-living microbes from larger organisms and particles.

### Clone library construction and sequencing

DNA was extracted from filters using the method described elsewhere [12] and PCR-amplified with the universal bacterial primers 27BF (5′-AGAGTTTGATCCTGGCTCAG-3′) and 907RAB (5′-TTTGAGTTTMCTTAACT GCC-3′) [13], using the following conditions: 5 min hot start at 94°C, followed by denaturation for 90 s at 94°C, annealing for 90 s at 50°C and 2 min of extension at 72°C. On the 25^th^ and final cycle, the extension time was increased to 5 min. The 16S rRNA gene libraries were constructed from the pooled PCR product using a pGEM-T easy vector system (Promega) and transformed into electrocompetent *Escherichia coli* DH10B cells according to the manufacturer's instructions. Transformants were selected by blue–white screening methods on Luria–Bertani agar supplemented with ampicillin (100 mg.ml^−1^) and X-gal (100 mg.ml^−1^). About 700 clones containing a putative 16S rRNA gene fragment were randomly selected and submitted to sequencing. Sequencing was performed using the MegaBace1000 DNA analysis system (GE Healthcare). DNA sequences were proofread and all ambiguities were removed. The program Mallard [14] was used to identify whether any chimeric sequences were present in the library. The representative sequences generated in this study were deposited in the GenBank under the accession numbers JN649376 to JN650045.

### Bioinformatic analysis

All sequences were globally aligned using the MUSCLE software [15] and further refined manually. Distance data were generated from the clone library using the Kimura two-parameter model and analyzed using the computer program MOTHUR [16] to group sequences into operational taxonomic units (OTU), based on a 97% sequence identity cutoff. MEGA4 [17] was used to construct a UPGMA tree, which was bootstrap resampled 1000 times. Unweighted Principal Coordinates Analysis (PCoA) was performed to evaluate similarity among samples using UNIFRAC [18].

## Results and Discussion

**Figure 1:**
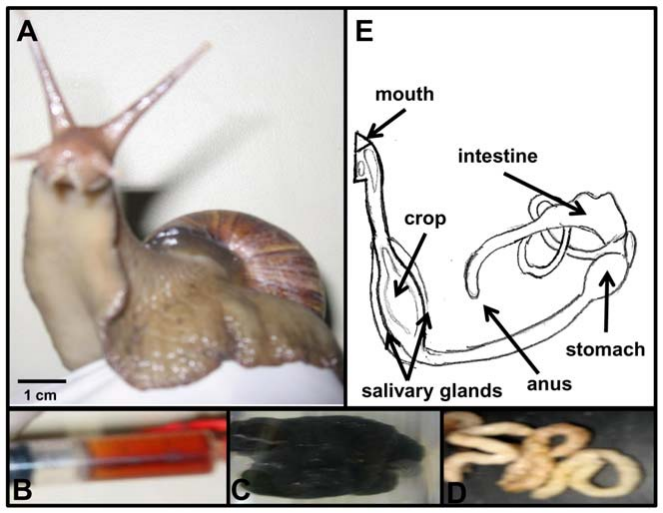
Large and healthy snail (A) and its crop fluid (B), feces from field (C) or reared (D) animals are shown. Schematic representation of the *Achatina* digestive tract (E).

Little is known about the composition of snail microbiota. In this work, we investigated whether the diversity and composition of bacterial communities varies along different parts of the digestive tract of the giant land snail *Achatina fulica*. We also compared the relative effect of a high carbohydrate diet on gut bacterial community structure. *A. fulica*'s gut is remarkably simple (Figure 1), possibly due to its terrestrial life and feeding habits. The crop is the largest part of the foregut and it is the site of storage and initial digestion of food [19]. The intestine is a long, narrow and coiled tube as in other herbivores [20]. To characterize the microbial populations in the snail's gastrointestinal tract, we sampled the bacterial contents within the crop (C) and the fecal samples (I) from seven field-collected snails (FC).

The experimental snails (RL) were allowed to ingest prepared food (sugarcane) twice a week. Once a week the containers were washed carefully with hot water. After 24 weeks of feeding under laboratory conditions the snails were left for 72 h without food to cleanse their digestive system, and then the crop fluid samples were collected. It is likely that after starvation the community in the digestive tract is reduced to stable members. To study intestinal microbial community composition in planorbid species, the snails were starved for 24 h prior to dissection [11]. In the gut of *Helix aspersa*, this time is sufficient to clear the cultivable bacteria of the transient gut flora [21]; however, further investigation into the bacterial structure of crop fluid from snails is recommended. In addition, recent findings suggest that there can be an adjustment in the microbial gut population 24 h after a change in diet that lasts for 10 days [22].

The snails were considered active and unaffected by this restricted diet when the amount of feces per animal per day did not decrease by >10% when compared to controls. Furthermore, no significant difference was observed in snail growth, but in contrast, the RL animals showed a drastic change in oviposition and did not produce eggs, probably due to nutrient and calcium deficiency (not shown). The crop samples in this study were obtained by cannulation of the mouth-oesophagus with a needleless scalp vein set attached to a syringe. By using cannulation and collecting fecal samples, no animal was killed for the purposes of this study. This method was sought to allow multiple sampling of the same snails over time. Such sampling procedure may provide researchers with a simple method to follow shifts in microbial community in land snails in future investigations.

**Figure 2:**
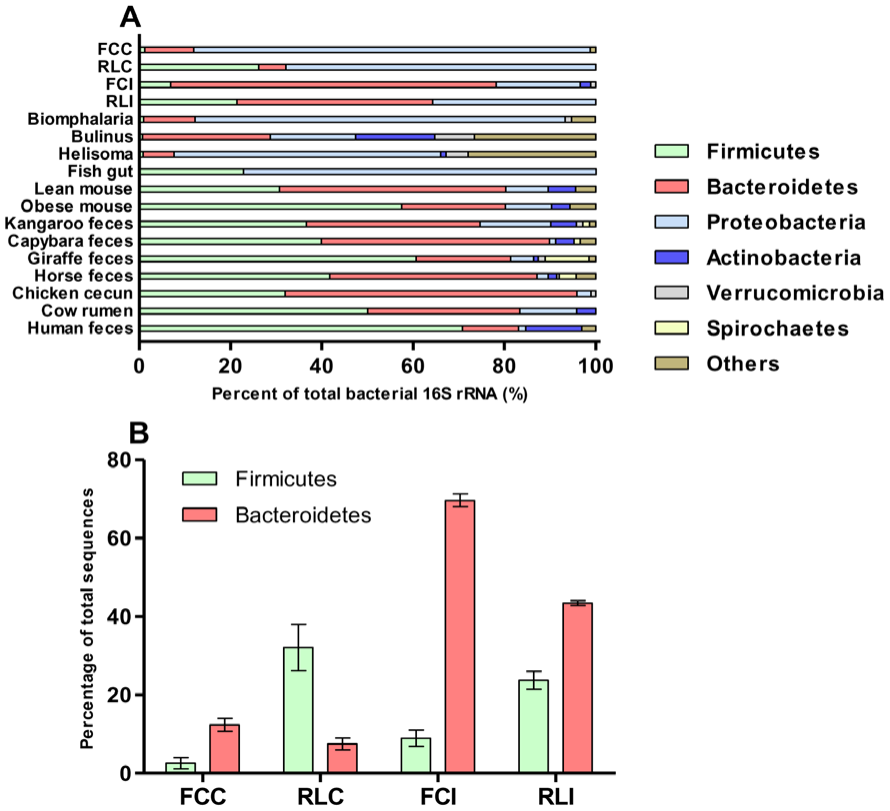
Distribution of sequences in bacterial phyla classified by the RDP Database (A) and proportion (B) of *Firmicutes* and *Bacteroidetes* in the crop and intestine (feces) microbiota of wild vs. reared snails. Clones were designated FC to indicate field-collected snails; RL, reared in the laboratory; C, crop; and I, intestine (feces). The datasets were compared against the following MG-RAST (metagenomics.anl.gov) metagenomic projects: Fish gut (4441695.3); Lean (4440463.3) and Obese Mouse (4440464.3); Red kangaroo (4461325.3); Capybara (4461352.3); Giraffe (4461358.3); Horse (4461321.3); Chicken cecum (4440285.3); Cow rumen (4441682.3) and Human (4440941.3). The sequences from planorbid snails *Biomphalaria pfeifferi* (FJ228890–FJ229104), *Bulinus africanus* (FJ228740–FJ228889), and *Helisoma duryi* (FJ229105–FJ229355) were obtained from NCBI dataset.

A total of 670 bacterial sequences with approximately 828 bp were analyzed by MOTHUR, yielding 228 operational taxonomic units (OTUs) grouped at 97% stringency. Rarefaction analysis showed that the *Achatina fulica* and planorbid snails [11] datasets afforded a similar degree of coverage of the biodiversity present in these microbiomes, showing higher bacterial species diversity within the snails (Figure S2). In order to reveal bacterial phyla composition, sequences from each library were classified with the RPD classifier tool [23] and compared with other gut bacteria within foregut, hindgut-fermenting herbivores and other animals (Figure 2).

**Figure 3:**
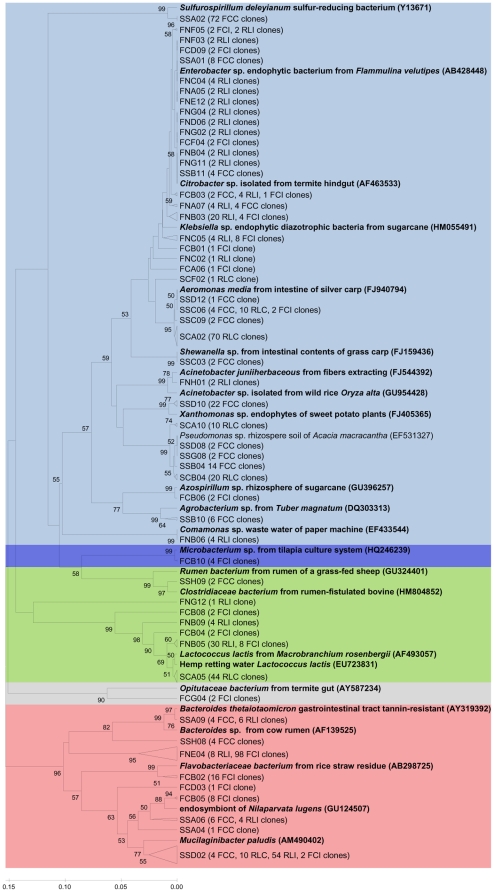
Phylogenetic tree of bacterial clones obtained within the snail gut. Reference sequences from GenBank (in bold). OTUs were defined by using a distance level of 3% by using the furthest neighbor algorithm in MOTHUR. One access number from each OTU is displayed. The tree topology is based on UPGMA method and was constructed in MEGA. The bootstrap analysis was performed with 1000 replications. Bootstrap value >50 and representative OTUs are shown. Clones were designated FC to indicate field-collected snails; RL, reared in the laboratory; C, crop; I, intestine (feces); followed by the clone number. Colored according to Figure 2.

Phylogenetic relationships of land snail clones with known bacterial isolates or environmental rDNA sequences were also analyzed (Figure 3). Crop fluid samples showed a higher abundance of *Proteobacteria* while fecal samples were dominated by *Bacteroidetes* and *Firmicutes*, abundant microorganisms in the feces of warm-blooded animals including humans. Within the *Epsilon-proteobacteria*, a profuse OTU with 72 sequences was related to *Sulforospirillum* spp. Interestingly, in the gutless marine oligochaete *Olavius algarvensis*, endosymbiotic sulphate-reducing bacteria serve as an energy source to the host and may participate within the host in the removal of the end products of fermentation [24]. Other bacterial taxa closely related to herbivore and plant-associated bacteria included *Clostridiaceae*, *Lactococcus*, *Bacteroides*, *Flavobacteriaceae*, *Mucilaginibacter*, *Citrobacter*, *Klebsiella*, *Aeromonas*, *Acinetobacter*, *Pseudomonas*, and *Comamonas* (Figure 3).

A shift in gut microbial communities takes place in snails consuming the sugarcane diet. The representation of the *Bacteroidetes* diminishes by 50%, and the *Firmicutes* increase to a corresponding degree when compared with field-collected snails (Figure 2). Remarkably, a similar shift in the ratio of *Bacteroidetes* to *Firmicutes* occurs in obese compared to lean mice [25], humans [26], and pigs [27]; in addition, there is a division-wide increase in the proportion of *Bacteroidetes* and reduction in *Firmicutes* as humans lose weight. Although this shift is not fully understood, it may control the energy balance equation in the host [26].

**Figure 4:**
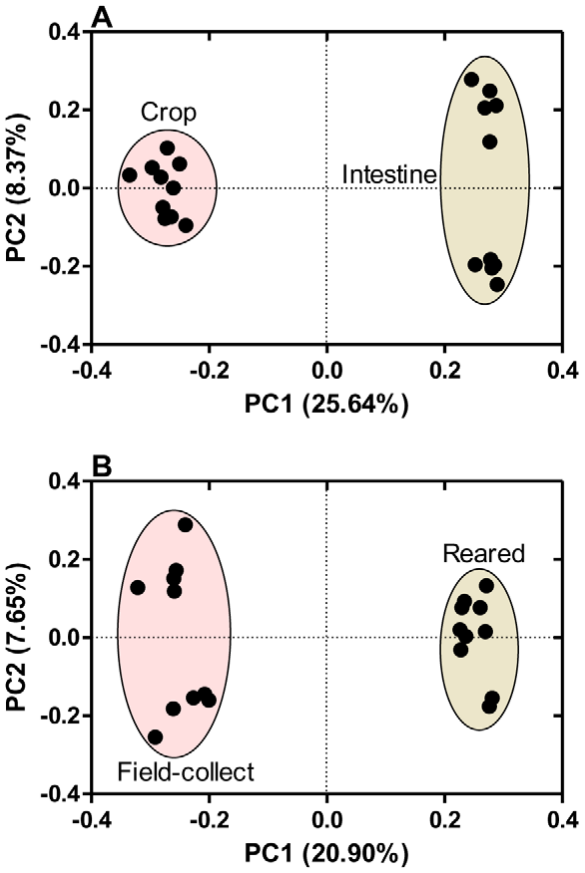
Match between bacterial communities comparing crop vs. feces (A) and wild vs. reared snails (B). Principal coordinates plots (PCoA) were generated using the pair wise unweighted UniFrac distances.

The clustering of gut bacteria by host diet (herbivore, omnivore, and carnivore) and phylogeny was highly significant in both the tree-based and network-based analyses [20], suggesting a direct link between diet and microbial community composition. Principal Coordinates Analysis plots (PCoA) generated using pairwise unweighted UniFrac distances (Figure 4) showed that the bacterial community structure of crop fluid was different from that of the feces. UniFrac clearly separated different microbiota efficiently not only by diet but also by gut type (crop and intestine). The clustering according to UniFrac is striking, suggesting the importance of food and anatomy as a driver of community composition in this terrestrial snail.

Recently, it was shown that the adaptation of the microbiota to diet is similar across different mammalian lineages [28] but in invertebrates this is still an open question and clearly more research is necessary to give better insights into the coevolution of hosts and their gut microbiota. *Achatina* is largely herbivorous but equally opts for dead insects and snails. It is tempting to speculate that this mollusk selects its microbiota to survive and adapt to different habitats, contributing to its great dispersion. In addition to characterizing the snail microbial communities, we identified changes induced by diet. In summary, our findings show novel snail–microbe associations and, furthermore, suggest that the variety of bacteria within the gut might promote a better adaptation of the host to different diet conditions. Understanding and revealing the snail gut microbiota might contribute to controlling the invasion of this exotic species and give further insights into the host-bacteria association.

## Supporting Information

Figure S1 **Crop sample collection.** Cannulation of the mouth-oesophagus with a needleless scalp vein set attached to a syringe.
(TIF)

Figure S2 **Rarefaction curves of OTUs clustered at 97%.** *Achatina fulica* bacterial sequences are compared to *Biomphalaria pfeifferi* (FJ228890–FJ228967), *Bulinus africanus* (FJ228813–FJ228889), and *Helisoma duryi* (FJ229273–FJ229355).
(TIF)
