# Supplementary figures and images for: Gut Bacterial Communities in the Giant Land Snail Achatina fulica and Their Modification by Sugarcane-Based Diet
Source: PLoS One. 2012 Mar 15;7(3):e33440. doi: 10.1371/journal.pone.0033440 (PMC3305317; doi:10.1371/journal.pone.0033440)

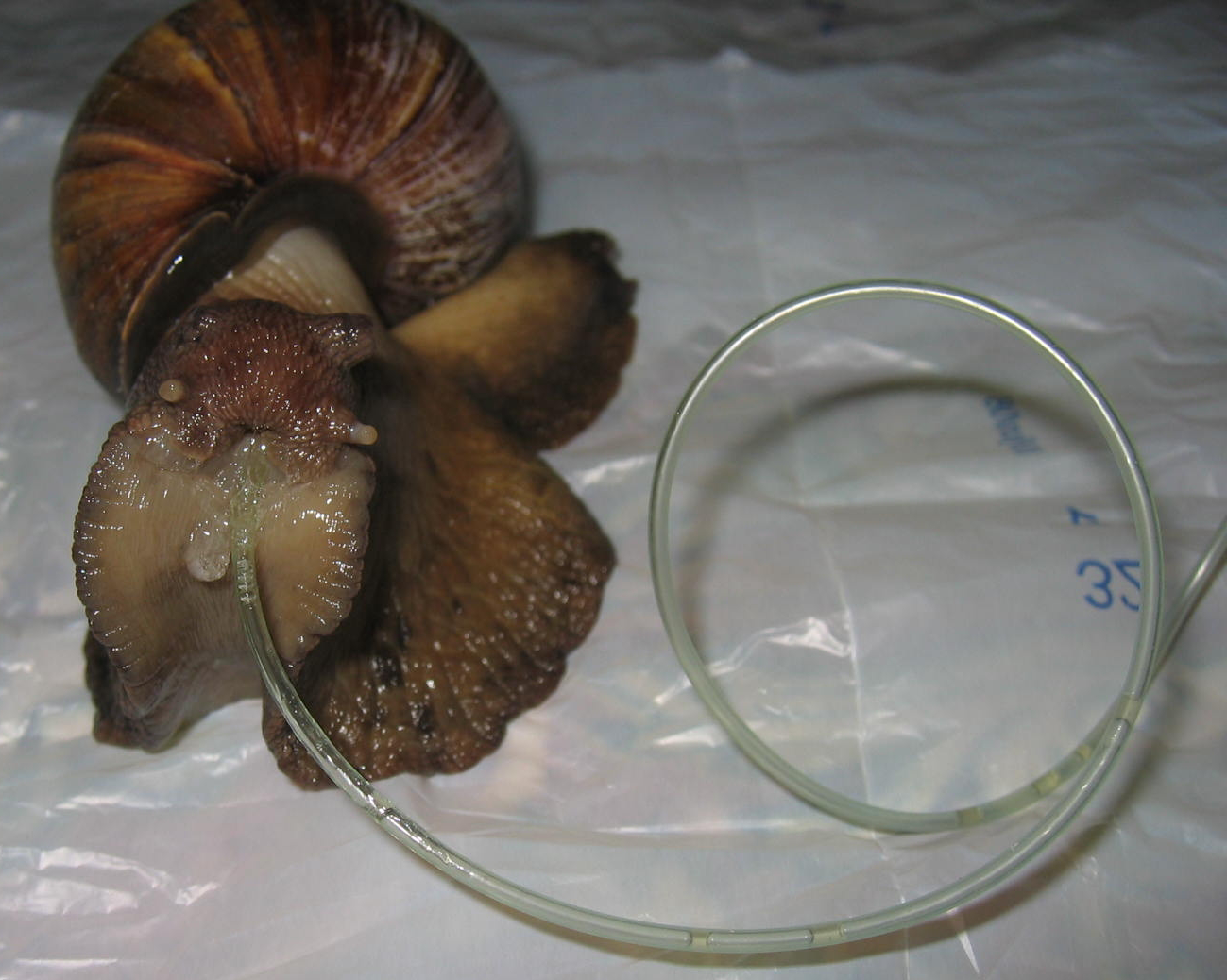

Supplement: Figure S1 — Crop sample collection. Cannulation of the mouth-oesophagus with a needleless scalp vein set attached to a syringe. (TIF) [file pone.0033440.s001.tif]

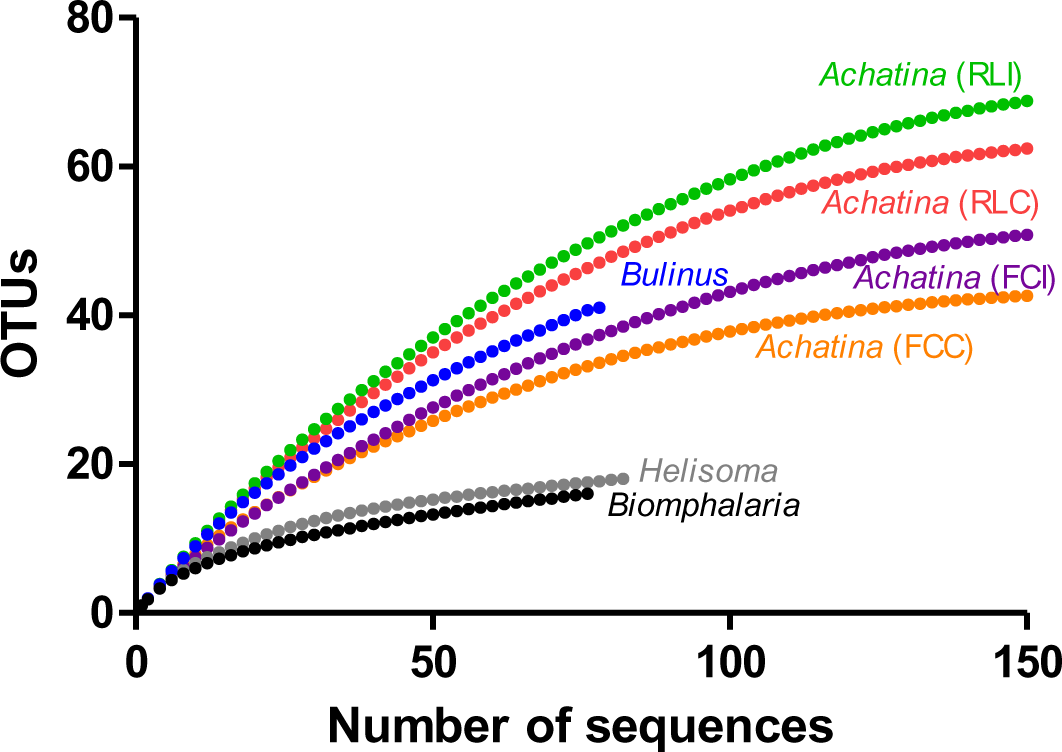

Supplement: Figure S2 — Rarefaction curves of OTUs clustered at 97%. Achatina fulica bacterial sequences are compared to Biomphalaria pfeifferi (FJ228890–FJ228967), Bulinus africanus (FJ228813–FJ228889), and Helisoma duryi (FJ229273–FJ229355). (TIF) [file pone.0033440.s002.tif]
